# Comparative Epidemiology and Resistance Trends of *Proteae* in Urinary Tract Infections of Inpatients and Outpatients: A 10-Year Retrospective Study

**DOI:** 10.3390/antibiotics8030091

**Published:** 2019-07-11

**Authors:** Márió Gajdács, Edit Urbán

**Affiliations:** 1 Department of Pharmacodynamics and Biopharmacy, Faculty of Pharmacy, University of Szeged, 6720 Szeged, Eötvös utca 6., Hungary; 2 Institute of Clinical Microbiology, Faculty of Medicine, University of Szeged, 6725 Szeged, Semmelweis utca 6., Hungary; 3 Department of Public Health, Faculty of Medicine, University of Szeged, 6720 Szeged, Dóm tér 10., Hungary

**Keywords:** urinary tract infection, UTI, antibiotic, resistance, indicator, epidemiology, fosfomycin, *Morganella*, *Proteus*, *Providencia*

## Abstract

Compared with infections caused by other bacterial pathogens, urinary tract infections (UTIs) caused by *Proteae* are often more severe and associated with a higher rate of recurrence, sequelae, and pyelonephritis. The aim of this retrospective study was to assess and compare the prevalence of UTIs caused by different species of the *Proteae* tribe (namely *Proteus*, *Morganella* and *Providencia* species) and the antibiotic resistance levels isolated from inpatients and outpatients in a primary- and tertiary-care teaching hospital in the Southern Great Plain of Hungary, during a 10-year study period. To evaluate the resistance trends of isolated strains, amoxicillin/clavulanic acid, ceftriaxone, meropenem, ertapenem, gentamicin, ciprofloxacin, and fosfomycin were chosen as indicator antibiotics, based on local antibiotic utilization data. Members of *Proteae* were more frequently isolated in the case of inpatients (7.20 ± 1.74% vs. 5.00 ± 0.88%; *p* = 0.0031), *P. mirabilis* was the most frequently isolated member of the group. The ratio of resistant strains to sulfamethoxazole/trimethoprim, ciprofloxacin, ceftriaxone, and fosfomycin was significantly higher in the inpatient group. In the case of amoxicillin/clavulanic acid, ceftriaxone, ciprofloxacin, and sulfamethoxazole/trimethoprim, the ratio of resistant isolates was markedly higher between 2013–2017 (*p* < 0.01). Resistance developments of *Proteae*, coupled with their intrinsic non-susceptibility to several antibiotics (tetracyclines, colistin, nitrofurantoin) severely limits the number of therapeutic alternatives, especially for outpatients.

## 1. Introduction

Urinary tract infections (UTIs) are one of the most common infections in both community (accounting for 10–30% of infections in primary care) and hospital settings (30–40%) [1,2,3]. In addition to their high incidence and diverse spectrum of etiological agents, these infections are often associated with recurrence, complications, and sequelae, corresponding to a decreased quality of life (QoL) for the affected patients [1,2,3,4,5]. For this reason, UTIs should be considered as an important factor of morbidity. The most common causative agents of UTIs are *Escherichia coli*, *Klebsiella* spp., *Enterococcus faecalis*, members of the *Proteae* tribe (see below), *Pseudomonas aeruginosa*, Group B streptococci (GBS), *Staphylococcus saprophyticus*, and *S. aureus*, in addition to *Candida* spp.; however, the distribution of etiological agents in UTIs have changed considerably, both in nosocomial and community settings [1,2,3,4,5,6]. Although there have been developments in novel antimicrobial drugs in the last decade, the treatment of UTIs is an increasingly complex challenge for clinicians [7]. The growing levels of antibiotic resistance (especially in Gram-negative bacteria) severely limits the available treatment options in these pathologies [8]. Nevertheless, with the emergence of extended-spectrum β-lactamase (ESBL) and carbapenemase-producing strains in urinary pathogens, multidrug resistance (MDR) is a growing concern because, when coupled with other inherent and acquired resistance mechanisms, these pathogens may be resistant to a broad range of antibiotics [9,10,11,12].

Most members of the *Morganellaceae* family (including genera *Arsenophonus*, *Cosenzaea*, *Moellerella*, *Morganella*, *Photorhabdus*, *Proteus*, *Providencia*, and *Xenorhabdus*) are peritrichous Gram-negative rods, that are ubiquitous in the environment [13,14,15]. Taxonomically, *Morganellaceae* are found in the order *Enterobacterales* (*Gammaproteobacteria*), which has been recently re-organized based on comparative genomic analyses [16]. All human pathogenic members of the *Morganellaceae* family are found in the *Proteae* tribe, comprising of the genera *Proteus* (including *P. hauseri*, *P. mirabilis*, *P. myxofaciens*, *P. penneri*, *P. vulgaris*, and several yet unnamed genospecies), *Morganella* (including *M. morganii subsp. morganii* and *M. morganii subsp. sibonii*), and *Providencia* (including *P. alcalifaciens*, *P. heimbachae*, *P. rettgerii*, *P. rustigianii*, and *P. stuartii*) [13,14]. Species of *Proteae* are widespread in the environment and are normal inhabitants of the gut microbiota, in addition, they are the third most frequent causative agents of UTIs, especially in nosocomial settings [2,3,4,17]. They are characterized by strong urease production from urine (lowering the pH, which leads to tissue damage, scarring, and stone formation, through the composition of struvite and apatite crystals via precipitation of Ca^2+^ and Mg^2+^ ions), additionally, they possess virulence factors crucial for the pathogenesis of UTIs (IgA protease, hemolysin, cytotoxins, and fimbriae) [13,14,17,18,19]. In contrast to *E. coli*, *Proteae* are more frequently isolated in patients with complicated UTIs (i.e., the presence of urinary catheters and functional or anatomical abnormalities) and are frequently associated with pyelonephritis, recurrence and prolonged treatment [20,21,22,23]. They are intrinsically resistant to several groups of antibiotics (nitrofurantoin, tetracyclines, and colistin; this intrinsic resistance may be used as a presumptive identification marker for these organisms), they produce various β-lactamases (penicillinases, AmpC-β-lactamases) and have an intrinsic reduced susceptibility to imipenem, making the management of these infections even more difficult [13,14,17,18,19,24]. It is generally accepted that *P. mirabilis* has the most advantageous resistance trends among the *Proteae* group, while *P. vulgaris* and *Morganella* species have somewhat higher, and *Providencia* species have the highest rates of resistance to the relevant antibiotics. Indeed, these differences are frequently used in routine microbiology laboratories for their phenotypic identification/differentiation [13,14,17,18,19,24].

Since the 2000s, several national and global (e.g., the SENTRY Antimicrobial Surveillance Program or the Study for Monitoring Antimicrobial Resistance Trends; SMART) surveillance reports have evaluated and published the resistance trends of various Gram-positive and Gram-negative bacteria [25,26,27]. Nevertheless, there are very few studies available that specifically investigated the epidemiology and resistance rates of UTIs caused by the tribe *Proteae* as a whole, despite a trend of increased resistance in these pathogens having been observed in recent years, with the emergence of ESBL-producing *Proteus* species as a particular concern [28,29,30]. The epidemiology and antibiotic susceptibility-patterns of urinary tract pathogens vary greatly by region, and, therefore, the assessment of local data is essential to evaluate trends over time and to reflect on the national situation compared to international data [31]. Additionally, knowledge of the relevant antibiotic susceptibility patterns of the major bacterial pathogens for UTIs is of utmost importance to allow for the optimal choice for antibiotic therapy [32,33,34]. 

With this in mind, the aim of this study was to assess and compare the prevalence of UTIs caused by different species of the *Proteae* tribe (namely *Proteus*, *Morganella* and *Providencia* species) from inpatients and outpatients and the antibiotic resistance levels isolated at the Albert Szent-Györgyi Clinical Center (Szeged, Hungary) retrospectively, during a 10-year study period.

## 2. Results

### 2.1. Demographic Characteristics, Sample Types

The median age of affected patients was 75 years (range: 0.5–98) in the inpatient group with a female-to-male ratio of 0.83; the age distribution of patients was the following: 8.56% 0–5 years, 6.03% 6–14 years, 18.36% 15–65 years, and 67.05% over 65 years of age. In the outpatient group, the median age was 57 years (range: 0.5–98) with a female-to-male ratio of 0.91; the age distribution of patients was the following: 26.19% 0–5 years, 9.87% 6–14 years, 23.42% 15–65 years, and 40.52% over 65 years of age. The difference in the age distribution of the two patient groups was statistically significant (*p* < 0.0001). Most (99.3%) of the samples received from outpatient clinics were voided (midstream) urine, while the majority (69.38%) from the inpatient departments were catheter-specimen urine; midstream urine (28.89%) and samples obtained through suprapubic bladder aspiration (1.73%) were less numerous. 

### 2.2. Distribution of Proteae among Inpatient and Outpatient Urine Samples

**Figure 1:**
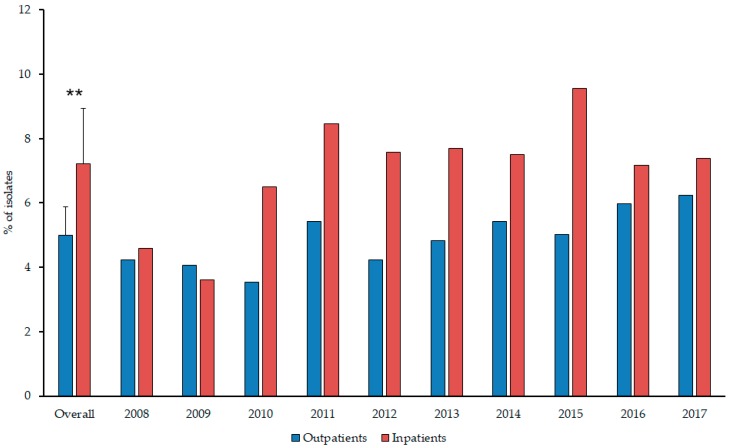
Ratio of *Proteae* in positive urine samples of inpatients and outpatients during the study period.

During the 10-year surveillance period (1st of January 2008–31st of December 2017), the Institute of Clinical Microbiology received 21,150 urine samples from outpatient clinics and 19,325 positive samples from inpatient departments that turned out to be positive for a significant urinary pathogen. From the outpatients, 1058 *Proteae* isolates were obtained and 1392 from the inpatients. Members of the *Proteae* tribe were more frequently isolated in the case of inpatients (7.20 ± 1.74% (range: 3.52–9.56%, lowest in 2009, highest in 2015) vs. 5.00 ± 0.88% (range: 3.55–6.25%, lowest in 2010, highest in 2017) of all positive urine samples; *p* = 0.0031) (Figure 1.). *P. mirabilis* was the most frequently isolated member of the group (inpatients: 81.54 ± 2.76% (range: 77.94–85.81%, lowest in 2008, highest in 2012); outpatients: 82.49 ± 4.76% (range: 74.63–92.11%, lowest in 2010, highest in 2012); *p* > 0.05), followed by *P. vulgaris* (inpatients: 13.24 ± 8.94% (range: 3.38–32.15%, lowest in 2012, highest in 2009); outpatients: 10.82 ± 6.45% (range: 3.95–23.88%, lowest in 2012, highest in 2010); *p* > 0.05), *M. morganii subsp. morganii* (inpatients: 5.07 ± 3.57% (range: 0–12.42%, lowest in 2011, highest in 2013); outpatients: 5.03 ± 3.29% (range: 0–9.23%, lowest in 2009, highest in 2015); *p* > 0.05) and *Providencia* spp., including *P. stuartii* and *P. rettgerii* (inpatients: 1.83 ± 2.72% (range: 0–8.39%, highest in 2017); outpatients: 0.91±0.89% (range: 0–2.63%, highest in 2016); *p* > 0.05). There were no statistically significant differences in the distribution of isolates species from inpatient and outpatient samples; however, the ratio of non-*Proteus* isolates was much higher in the second half (2013–2017) of the study period in both patient groups (*p* = 0.018 and *p* = 0.029, respectively). In 7.28% of inpatients and 8.33% of outpatients, co-infection with another urinary pathogen was detected (Table 1.), mainly associated with patients over 65 years of age and urinary catheters (in the case of inpatients). The distribution of co-isolated pathogens corresponds to the most frequently isolated bacteria in UTIs (e.g., *E. coli*, *K. pneumoniae*, *E. faecalis*). Co-pathogens were not found in urine samples with *Providencia* spp.

### 2.3. Antibiotic Susceptibility Trends among Proteae

**Figure 2:**
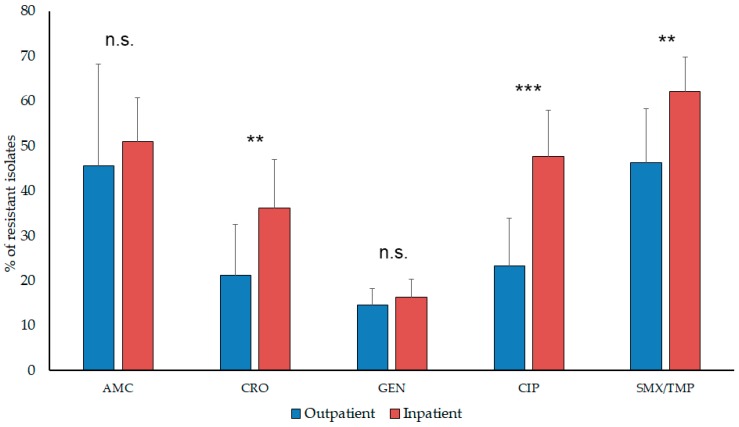
Resistance levels of *Proteae* originating from inpatient and outpatient urinary tract infections AMC resistance levels correspond to *P. mirabilis* isolates only.

The resistance trends of isolated *Proteae* for amoxicillin/clavulanic acid (AMC) (in the case of *P. mirabilis* only), ceftriaxone (CRO), gentamicin (GEN), ciprofloxacin (CIP), and sulfamethoxazole-trimethoprim (SXT) during the 10-year surveillance period are presented in Table 2. and Figure 2. The ratio of resistant strains in the inpatient group was significantly higher to SXT, CIP, and CRO (*p* = 0.0025, *p* < 0.0001, and *p* = 0.0071, respectively), but not in the case of GEN (*p* > 0.05). In contrast, AMC resistance in *P. mirabilis* was numerically, but not significantly higher (*p* = 0.499) in outpatient samples (going as high as 63.51–75.68% in the second half of the study period, while this ratio was 49.03–63.23% in the inpatient group). No statistical or numerical differences were observed among the resistance rates of various *Proteae* species (i.e., *Proteus* vs. *Providencia* vs. *Morganella; p > 0.05*).

The high incidence of strains resistant to ceftriaxone (>30% of isolates in the inpatient samples, while ranging between 17.76–38.14% for outpatient samples in the second half of the study period) peaked in 2011 (48.70%) has been consistent since 2010. Isolates originating from outpatient departments showed similar growing trends in the survey period. Ciprofloxacin resistance peaked in 2011 for the inpatient group (62.34%) and has remained above 40% since 2010; while, in the outpatient group, the peak occurred in 2015 (37.69%). The highest levels of resistance overall were recorded for SXT: Resistance in inpatient isolates peaked in 2011 (74.68%) and has never gone below 50% in the inpatient group; in the outpatient group, the peak occurred in 2015 (59.23%) and resistance levels were around 50% since 2011.

The temporal nature of resistance development could also be identified between the first (2008–2012) and second (2013–2017) half of the study period (*p* < 0.01) in the case of AMC, CRO CIP, and SXT, while the resistance levels of GEN has remained the lowest and relatively constant (compared to the other tested drugs, with 1.5–1.8-fold variation between the baseline and peak resistance) in the 10-year surveillance period.

No meropenem- or ertapenem-resistant isolates were recovered during the 10-year study period. Fosfomycin (FOS) susceptibility testing was performed for 17.77% of outpatient and 21.23% of inpatient isolates, respectively. Overall, the ratio of resistant strains was 30.33% (outpatients) and 18.69% (inpatients) (*p* = 0.023).

## 3. Discussion

Compared with UTIs caused by other relevant bacterial pathogens, UTIs caused by *Proteae* are often more severe and associated with a higher incidence of recurrence, sequelae, and pyelonephritis [13,14]. In addition, the majority of bloodstream infections caused by *Proteae* members originate from a UTI (urosepsis), frequently associated with urinary catheters and underlying conditions [35]. Increased resistance levels to antimicrobial agents are not only leading to changes in treatment guidelines and issues in the clinic, but also to poor prognoses, decreased QoL and an increase in the mortality rate of patients, especially in nosocomial settings [1,2,3,4,5,6]. Treatment of *Proteae* infections is especially challenging due to the various intrinsic resistance mechanisms they possess [13,14]. Other last-resort drugs used in severe infections with Gram-negative pathogens, like tigecycline and colistin are not useful in the therapy of *Proteae*-associated pathologies [36,37]. Several reports on the emergence and spread MDR *Proteus* and related species have also been published, which is further cause for concern [38,39]. This study presents the changing epidemiology and resistance trends of *Proteae* associated with urinary tract infections (UTIs) in Hungary over a long surveillance period (10 years), demonstrating a steady increase in the resistance levels regarding various antibiotics. To the best of our knowledge, this is the first and longest-spanning study reporting on the prevalence and susceptibility patterns of *Proteae* (and UTIs caused by these uropathogens by proxy) in Hungary.

The members of the *Proteae* tribe were causative agents in UTIs in around 5% of cases for outpatient and 7% in inpatient settings, and, therefore, their clinical relevance should not be disregarded [13,14,35]. Based on the results of this retrospective survey, the most prevalent isolate at our tertiary-care center remains *P. mirabilis*; however, a noteworthy increase (~5–7-times higher) was observed in the isolation rate of *Morganella* and *Providencia* species in the second half of the study period. Their higher prevalence in hospitalized patients and catheter-associated infections, a slight male dominance (~1.1–1.5-times) and the advanced age (over 65 years of age) of many affected patients is in line with the findings in the literature [13,14,17,18,19,24]. There is great variation in the species-distribution and susceptibilities of urinary tract pathogens in various parts of the globe, and, therefore, continuous and strict surveillance is recommended [40]. Based on relevant studies in the same geographical region (Southern Great Plain of Hungary), members of *Proteae* (especially *P. mirabilis*) are the third most commonly isolated microorganisms (*E. coli* and *Klebsiella* spp. were the most common, while after *Proteae*, non-fermenting Gram-negative bacteria, members of the CES group (*Citrobacter-Enterobacter-Serratia*) and *Candida* spp. were the most prevalent) [41,42]. In another study from Hungary (concentrating on the region near the capital) *Proteae* were isolated from 3–9% of urine samples; the resistance rates of these pathogens were the following: fluoroquinolones 10–50%, aminoglycosides 0–38%, ceftriaxone 0–9%, SMX/TMP 20–53%, and fosfomycin 0–14% [31]. Global reports suggest that overall, *P. mirabilis* is the fourth most common pathogen in UTIs, with an estimated prevalence of 2–2.8% [3]. The global SMART study, published by Morrissey et al., reported *P. mirabilis* in 3.6% of isolates (fourth most common); in this study, the ESBL-positivity for these bacteria were ~8% in the US, ~12% in Europe and >40% in some parts of Asia [25]. In a separate arm of the SMART study in Latin America, Ponce-de-Leon et al. reported 2% of *Proteus* isolates as ESBL-positive, while ceftriaxone, levofloxacin, and amikacin susceptibilities were 92%, 89%, and 100%, respectively [27]. In a study by Stefaniuk et al. in Poland, this was further verified, as the reported prevalence of *Proteus* spp. was 7.6% (2.8% in uncomplicated infections, while 13.5% in complicated UTIs), with a similar pattern of resistance trends as in the present study [5]. In a study from New York State, Rank et al. reported *Proteae* as the group fourth most common uropathogens, with the following susceptibility rates: SMX/TMP 87.0%, ciprofloxacin 84.0%, ceftazidime 93.0%, and tobramycin 95.0% [43]. In contrast, Yang et al. reported *Proteae* as the third most common UTI pathogen group in a tertiary-care hospital in China; the resistance rates of these isolates were 8.6–9.8% for amikacin, 12.1–14.6% for levofloxacin [33]. 

The role of the diagnostic bacteriology laboratories is to supply clinically relevant information in a precise and timely manner, which should be reciprocated by the feedback of the physicians, starting from the sample submission, followed by information regarding the symptoms of the patient and the clinical presentation [44]. Our local resistance results reflect the global pattern of increasing drug resistance in *Proteae* towards a variety of antimicrobial agents. It also highlights the importance of continuous surveillance at a local level in order to guide treatment recommendations for local use [43]. The results of this study suggest that—in most cases—empiric therapy for *Proteae* UTIs is not recommended, as the resistance rates (both for inpatient and outpatient samples) was over 20% for all of the tested drugs. Based on international guidelines, empiric antibiotic therapy is not recommended with a drug, where local resistance rates exceed 20% (in some cases 10%) [24,45]. In addition, the consultation with a hospital cumulative antibiogram (if it exists) is of the utmost importance, before the final choice of antimicrobial therapy [46]. The resistance developments of *Proteae*, coupled with their intrinsic non-susceptibility to several drug groups, severely limits the number of therapeutic alternatives, especially for outpatients. As a general rule, fosfomycin (if susceptibility is confirmed) still represents a safe and viable alternative for the therapy of these infections [47].

The indole-negative *P. mirabilis* strains are generally more susceptible to the relevant antibiotics than *P. vulgaris* (and other related species), *Morganella* spp., and *Providencia* spp [13,14,17,18,19,24]; however, this difference in resistance rates was not demonstrated in our present study. Gentamicin resistance has remained relatively constant in the surveillance period, while other antibiotics, which are more frequently used in primary care (especially amoxicillin/clavulanic acid, ciprofloxacin, sulfamethoxazole/trimethoprim, and fosfomycin) or are available in oral formulations, resistance levels show a pronounced and statistically significant increase [48]. Resistance rates for amoxicillin/clavulanic acid in *P. mirabilis* were two times higher in inpatient and six times higher in outpatient samples from baseline resistance values. Ciprofloxacin resistance rates were two times higher in inpatients and 4.5 times higher in outpatient from baseline resistance levels. Similarly, high levels of fluoroquinolone resistance have been reported for *Proteae* in regions where there are no restrictions and they are still considered to be first-line agents [49,50,51,52]. Quantitatively, antibiotic utilization levels in Hungary are relatively good; however, the qualitative analysis reveals a much bleaker picture: The use of broad-spectrum antimicrobials (including fluoroquinolones) is significantly higher, which may correspond to the development of local resistance trends [48,53,54]. Interestingly, while the ratio of resistant isolates from inpatient samples was significantly higher, the increase in the ratio of resistant strains over time was actually more pronounced in the isolates from outpatient samples. 

Based on the results of our study, the most concerning development is the resistance rates to third-generation cephalosporins (during our study ceftriaxone was chosen as an indicator; however, the group also includes cefotaxime, ceftriaxone, ceftazidime, and cefoperazone) [55]. Ceftriaxone-resistance rates have increased by three times in inpatient samples, and by almost ten times in outpatient samples. Resistance to β-lactam antibiotics is a severe issue in *Proteae* infections, especially in vulnerable patient groups (e.g., pregnant women, children), where some other therapeutic alternatives are inappropriate due to their teratogenicity. Resistance to third-generation cephalosporins (16.18–48.70% for inpatients and 4.05–38.14% for outpatients) in *Proteae* is mainly mediated through the production of ESBLs or AmpC β-lactamases [13,14,17,18,19,24]. Until recently, the detection of extended-spectrum beta-lactamase (ESBL)-producing isolates in *Proteae* was only recommended from blood culture isolates, and for public health purposes by EUCAST; however, this recommendation has been revised in 2017 (http://www.eucast.org/resistance_mechanisms/). Since this study reports on the resistance trends of *Proteae* between 2008–2017, the exact (molecular) identification of the β-lactam (i.e., ceftriaxone) resistance mechanism was, therefore, not performed during the study period. Additionally, this task was mainly carried out by a reference laboratory in Hungary for monetary considerations, and for invasive isolates only. Although the exact ratio of ESBL-positive CRO-resistant strains is unknown, there is literature data available, reporting that the ESBL-positivity rates are one of the lowest in *Proteae* (*Klebsiella* > *Escherichia* > *Enterobacter* > *Proteus* > *Morganella* > *Providencia*) [56]. In Hungary, since the 2000s, the most prevalent (>95%) type of ESBL-enzymes are the *bla*_CTX-M_-type beta-lactamases, which practically ousted most of the other types of ESBL-enzymes [57].

When it comes to carbapenems, it is well-known that imipenem is only marginally effective against *Proteae*. No meropenem-resistant strains were detected, therefore, in an inpatient setting (or through the utilization of outpatient parenteral antimicrobial therapy; OPAT) meropenem may be a safe option for now [58]. However, hospitalization in itself also carries a number of risks (e.g., acquiring nosocomial infections), especially in severely debilitated, immunocompromised patients. Nevertheless, this selection pressure will probably aid the emergence and global spread of carbapenemase-producing *Enterobacteriaceae* (CPE) even further, adding to the crisis of antimicrobial resistance [10]. However, if global resistance developments are any indication, Hungary will not avoid the increasing prevalence of MDR *Proteae*. 

Some limitations of this study must be acknowledged. Firstly, the design of this study is retrospective and, due to the inability to access the medical records of the individual patients affected, the correlation between the existence of relevant risk factors and underlying illnesses (apart from age, inpatient/outpatient status, and catheterization) and *Proteae* UTIs could not be assessed. The age-associated incidence in isolation of *Proteae* may also reflect (at least in part) the high rate of bacteriuria in the elderly population. Furthermore, phenotypic verification of the causes of β-lactam (ceftriaxone) resistance (AmpC- or ESBL-enzymes, porin loss, changes in permeability or other) or the molecular characterization of the genetic background of resistance (by PCR or sequencing) in the individual isolates was not performed, due to financial constraints, and previous local guidelines. There is a risk of selection bias, as most studies describing the prevalence of infectious diseases are tertiary-care centers, which generally corresponds to patients with more severe conditions or underlying illnesses. 

## 4. Materials and Methods 

### 4.1. Study Design, Data Collection

**Figure 3:**
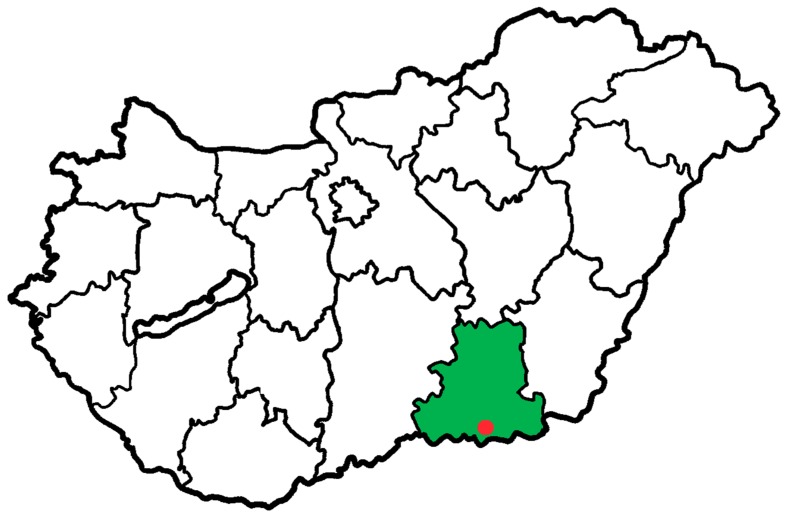
Study site in Hungary (Southern Great Plain of Hungary: in green; Albert Szent-Györgyi Clinical Center, Szeged: in red).

This retrospective study was carried out using microbiological data collected from the period between the 1st of January 2008 and 31st of December 2017 at the Albert Szent-Györgyi Clinical Center, a university-affiliated (University of Szeged), primary- and tertiary-care teaching hospital in the Southern Great Plain of Hungary. The Clinical Center has a bed capacity of 1820 beds (1465 active and 355 chronic beds, respectively) and annually serves more than 400,000 patients in the region (based on the data of the National Health Insurance Fund) (Figure 3) [59]. An electronic search in the records of the MedBakter laboratory information system (LIS) for urine samples submitted with the suspicion of a urinary tract infection was conducted by the authors (M.G. and E.U.). Samples with clinically significant colony counts for *Proteae* (10^5^< CFU/mL; however, this was subject to interpretation, based on the information provided on the request forms for microbiological analysis and relevant international guidelines) were included in the data analysis. Isolates were considered separate if they occurred more than 14 days apart or isolates with different antibiotic-susceptibility patterns were detected [60]. In addition, patient data limited to demographic characteristics (age and sex) were also collected.

### 4.2. Identification of Isolates

10 µL of each un-centrifuged urine sample was cultured on UriSelect chromogenic agar plates (Bio-Rad, Berkeley, CA, USA) with a calibrated loop, according to the manufacturer’s instructions and incubated at 37 °C for 24–48 hours, aerobically. If the relevant pathogens presented in significant colony count, the plates were passed on for further processing. Between 2008–2012, presumptive phenotypic (biochemical reaction-based) methods and VITEK 2 Compact Automated ID/AST System (bioMérieux, Marcy-l'Étoile, France) were used for bacterial identification, and, after 2013, this was complemented by matrix-assisted laser desorption/ionization time-of-flight mass spectrometry (MALDI-TOF MS; Bruker Daltonik Gmbh. Gr.). The methodology of sample preparation for MALDI-TOF MS measurements was described elsewhere [61]. Mass spectrometry was performed by the Microflex MALDI Biotyper (Bruker Daltonics, Germany) in positive linear mode across the m/z range of 2 to 20 kDa; for each spectrum, 240 laser shots at 60 Hz in groups of 40 shots per sampling area were collected. The MALDI Biotyper RTC 3.1 software (Bruker Daltonics, Germany) and the MALDI Biotyper Library 3.1 were used for spectrum analysis.

### 4.3. Antimicrobial Susceptibility Testing

Antimicrobial susceptibility testing (AST) was performed using the Kirby–Bauer disk diffusion method and E-test (Liofilchem, Abruzzo, Italy) on Mueller–Hinton agar (MHA) plates. In addition, for the verification of discrepant results, VITEK 2 Compact Automated ID/AST System (Gr- AST card) (bioMérieux, Marcy-l'Étoile, France) was also utilized. The interpretation of the results was based on EUCAST breakpoints (http://www.eucast.org). *Staphylococcus aureus* ATCC 29213, *Enterococcus faecalis* ATCC 29212, *Proteus mirabilis* ATCC 35659, *Escherichia coli* ATCC 25922 and *Pseudomonas aeruginosa* ATCC 27853 were used as quality control strains.

To evaluate the resistance trends of isolated strains, amoxicillin/clavulanic acid (AMC), ceftriaxone (CRO), meropenem (MER), ertapenem (ERT), gentamicin (GEN), ciprofloxacin (CIP), sulfamethoxazole/trimethoprim (SXT), and fosfomycin (FOS) were chosen as indicator antibiotics, based on local antibiotic utilization data [62,63,64,65]. AMC resistance data was collected for P. mirabilis only (as most other members of Proteae are intrinsically resistant) [14]. Susceptibility testing data for fosfomycin (FOS) was available from 2012; FOS susceptibility testing was not routinely performed, only in cases of extensive drug resistance or per request of the clinicians [66]. During data analysis, intermediately-susceptible results were grouped with and reported as resistant.

### 4.4. Statistical Analysis

Descriptive statistical analysis (including means or medians with ranges and percentages to characterize data) was performed using Microsoft Excel 2013 (Redmond, WA, Microsoft Corp.). Statistical analyses were performed with SPSS software version 24 (IBM SPSS Statistics for Windows 24.0, Armonk, NY, IBM Corp.), using the χ^2^-test, Student’s t-test and Mann–Whitney U test. The normality of variables was tested using Shapiro–Wilk tests. P values < 0.05 were considered statistically significant.

## Figures and Tables

**Table 1:** Frequency of co-isolation and species distribution in inpatient and outpatient samples.

Isolated co-pathogen	Setting	*Proteus* spp.	*Morganella* spp.
*Escherichia coli*	*Inpatient*	41	5
	*Outpatient*	29	3
*Enterococcus faecalis*	*Inpatient*	31	3
	*Outpatient*	22	2
*Klebsiella pneumoniae*	*Inpatient*	23	1
	*Outpatient*	7	2
*Pseudomonas aeruginosa*	*Inpatient*	8	0
	*Outpatient*	9	0
*Enterobacter aerogenes*	*Inpatient*	2	0
	*Outpatient*	2	0
*Candida albicans*	*Inpatient*	0	0
	*Outpatient*	2	0
*Acinetobacter baumannii*	*Inpatient*	0	0
	*Outpatient*	0	1

**Table 2:** Percentage of resistant strains to indicator antibiotics from inpatient and outpatient departments (2008–2017) ^a^.

Study Period		2008	2009	2010	2011	2012	2013	2014	2015	2016	2017	Statistics
**AMC R (%) ^b^**	*Outpatient*	*12.16*	17.57	31.08	60.81	28.38	37.84	63.51	66.22	**75.68**	62.16	n.s. (*p* = 0.499)
	*Inpatient*	*32.35*	37.50	47.17	59.74	56.08	**63.23**	54.44	56.56	53.13	49.03	
**CRO R (%)**	*Outpatient*	*4.05*	4.11	11.94	27.72	31.58	**38.14**	25.41	26.15	17.76	24.10	*P* = 0.0071
	*Inpatient*	*16.18*	23.21	33.02	**48.70**	47.30	47.10	41.42	40.27	33.75	30.97	
**GEN R (%)**	*Outpatient*	*10.81*	13.70	20.90	10.89	10.53	11.34	15.57	**18.46**	15.79	17.47	n.s. (*p* = 0.32)
	*Inpatient*	16.18	14.29	16.04	11.69	20.27	14.19	*10.06*	**23.53**	18.75	18.06	
**CIP R (%)**	*Outpatient*	*8.11*	10.96	11.94	23.76	30.26	18.56	27.05	**37.69**	26.97	37.35	*P* < 0.0001
	*Inpatient*	35.29	*26.79*	45.28	**62.34**	56.76	50.97	52.07	43.89	44.38	40.00	
**SXT R (%)**	*Outpatient*	*28.38*	35.62	32.84	45.54	50.00	38.14	59.02	**59.23**	57.24	57.23	*P* = 0.0025
	*Inpatient*	55.88	*50.00*	50.94	**74.68**	68.24	61.94	66.27	64.71	62.50	65.16	

^a^ Values in *italics* represent the lowest (baseline) resistance levels, boldface (peak) values correspond to the highest resistance levels in the study period; ^b^ Corresponding to *P. mirabilis* susceptibility; n.s.: not significant.

## List of abbreviations

**AMC:** amoxicillin/clavulanic acid; **AST:** antimicrobial susceptibility testing; **CFU:** colony-forming unit; **CPE:** carbapenem-resistant *Enterobacteriaceae*; **CIP:** ciprofloxacin; **CRO:** ceftriaxone; **ESBL:** extended-spectrum β-lactamase; **EUCAST:** European Committee for Antimicrobial Susceptibility Testing; **FOS:** fosfomycin; **GBS:** Group B *Streptococcus*; **GEN:** gentamicin; **ID:** identification; **LIS:** laboratory information system; **MALDI-TOF MS:** matrix-assisted laser desorption/ionization time-of-flight mass spectrometry; **MDR:** multidrug-resistance; **OPAT:** outpatient parenteral antibiotic therapy; **PCR:** polymerase chain reaction; **QoL:** quality-of-life; **SXT:** sulfamethoxazole-trimethoprim; **UTI:** urinary tract infection
